# A case control study of sarcosine as an early prostate cancer detection biomarker

**DOI:** 10.1186/s12894-015-0095-5

**Published:** 2015-10-01

**Authors:** Donna P. Ankerst, Michael Liss, David Zapata, Josef Hoefler, Ian M. Thompson, Robin J. Leach

**Affiliations:** Department of Mathematics, Technische Universitaet Muenchen, Boltzmannstr 3, Garching, 85748 Germany; Department of Urology, University of Texas Health Science Center at San Antonio, 7703 Floyd Curl Dr, San Antonio, TX 78229 USA; Department of Epidemiology and Biostatistics, University of Texas Health Science Center at San Antonio, 7703 Floyd Curl Dr, San Antonio, TX 78229 USA; Department of Cellular and Structural Biology, University of Texas Health Science Center at San Antonio, 7703 Floyd Curl Dr, San Antonio, TX 78229 USA

**Keywords:** Sarcosine, Prostate cancer, Prostate-specific antigen

## Abstract

**Background:**

Sarcosine has been investigated as a prostate cancer biomarker with mixed results concerning its predictive power. We performed a case–control evaluation of the predictive value of serum sarcosine for early detection in a population-based cohort of men undergoing prostate-specific antigen (PSA) screening.

**Methods:**

For analysis we used 251 cancer cases and 246 age-matched non-cancer cases from the San Antonio Biomarkers Of Risk (SABOR) screening study. For cancer cases, pre-diagnostic serum was utilized for sarcosine measurement. Controls were defined as men who had been followed at least for 5 years on study with no prostate cancer diagnosis; sarcosine was measured on the initial baseline serum. HPLC-electrospray ionization mass spectrometry was used for serum sarcosine quantification. The association of sarcosine with prostate cancer was assessed using area underneath the receiver-operating characteristic curve (AUC), and logistic regression adjusting for PSA, digital rectal exam, family history, age, race, and history of a prior negative biopsy. Among cancer cases, nominal logistic regression was used for the association of sarcosine with Gleason grade.

**Results:**

Sarcosine levels were overlapping between the prostate cancer cases (median 15.8 uM, range 6.2 to 42.5 uM) and controls (median 16.2 uM, range 6.4 to 53.6 uM). The AUC of sarcosine was not statistically different from random chance either for participants with any PSA value (52.2 %) or those with PSA values in the range of 2 to 10 ng/mL (54.3 %). Sarcosine was not predictive of Gleason score and added no independent predictive power to standard prostate cancer risk factors for detection of prostate cancer (all *p*-values > 0.05).

**Conclusions:**

Serum sarcosine should not be pursued further as a marker for the early detection of prostate cancer.

## Background

Metabolic profiling has identified sarcosine as a differential metabolite in the development and progression of prostate cancer [1]. Sarcosine, also known as N-methyglycine, is an N-methyl derivate of the amino acid glycine synthesized by glycine-N-methyltransferase (GNMT). GNMT has been implicated in methylation and prostate cancer [2]. This link led to interest in sarcosine as a prostate cancer biomarker. In the few prior studies investigating sarcosine as a biomarker of prostate cancer, results have been conflicting; some indicated higher risk associated with higher levels of sarcosine and others, a lower risk [3, 4]. The methodology of these studies has been either unclear or suboptimal, for instance by using serum samples far in advance of the time of biopsy. In order to definitively assess the potential of serum sarcosine for further study, we evaluated its operating characteristics and independent predictive value in a prospectively collected cohort of men, specifically assembled for the evaluation of prostate cancer biomarkers.

## Methods

Serum samples were obtained spanning the years from 2001 to 2009 from men enrolled in a prospective cohort undergoing annual prostate-specific antigen (PSA) testing as part of the San Antonio Biomarkers Of Risk (SABOR) clinical validation site for the US National Cancer Institute Early Detection Research Network (EDRN). The study protocol and written consent procedures were approved by the institutional review board at the University of Texas Health Science Center at San Antonio (UTHSCSA). Cases, controls, serum samples, and risk measures were selected as previously described [5]. Serum specimens were collected within 2.5 years prior to the prostate cancer diagnosis for cases and at the first baseline visit for controls; the majority of specimens (92.7 %) were within 1 year. Controls included cancer-free men with a minimum of 5 years follow-up after serum collection, a group selected to minimize the risk of assigning as a control a subject who harbored a tumor that would be discovered within the intermediate term. The risk factors measured included PSA (measured from the same serum sample as used for sarcosine), digital rectal exam (DRE, result used from the same patient visit as that for serum measurement of PSA), age, race, family history of prostate cancer in a first-degree relative, and history of a prior negative biopsy.

Serum sarcosine was measured using the UTHSCSA and Cancer Center Mass Spectrometry Core Facility. Serum (20 μl) was combined with stable isotope labeled internal standard [2H3] sarcosine (25 μM, 40 μl), water (40 μl), and sodium tetraborate solution (0.1 M, 50 μl) prior to derivatization with 5-(dimethylamino) naphthalene-1-sulfonyl chloride (dansyl chloride; 20 mM, 100 μl) at room temperature for 30 min. After evaporation to dryness by vacuum centrifugation, the derivatized samples were dissolved in 200 μl of mobile phase A and transferred to autosampler vials for HPLC-ESI-MS analysis on a Thermo Fisher Q Exactive mass spectrometer used in conjunction with a Thermo Fisher/Dionex Ultimate 3000 HPLC. Peak areas were determined by processing through Quan Browser (Thermo Fisher) and compared to calibration curves that were generated by analysis of authentic standards.

Risk factors and serum sarcosine levels were compared between cases and controls using the chi-square test for categorical outcomes and Wilcoxon test for continuous measures. The AUC was computed for sarcosine and tested using the Wilcoxon rank test for all cases and controls, and in addition restricted to the group of cases and controls with PSA in the 2 to 10 ng/mL range. Logistic regression was used to assess the independent predictive power of sarcosine to PSA, DRE, age, race, history of a prior negative biopsy, and family history of prostate cancer. Among prostate cancer cases, multinomial logistic regression was used to assess whether sarcosine was associated with the trivariate outcome of no cancer, low-grade cancer (Gleason score < 7), and high-grade cancer.

## Results

Characteristics of the 251 prostate cancer cases and 246 controls participating in the SABOR screening study are shown in Table 1. Participants diagnosed with prostate cancer were more likely to be of other races than Caucasian, have an abnormal DRE, have a positive family history of prostate cancer, and have a higher PSA value (all *p* < 0.05). Controls had similar ages and history of prior negative biopsy to the cancer cases.

**Table 1:** SABOR case control demographic and clinical variables

	Control	Prostate cancer	
	Median (range)	Median (range)	
	or *N* (%)	or *N* (%)	*P* value
	*n* = 251	*n* = 246	
Age	64.8 (45.0–83.5)	64.1 (46.0–88.5)	0.80
Race			0.02
White	171 (68.1)	139 (56.5)	
African American	34 (13.5)	38 (15.4)	
Other	46 (18.3)	69 (28.0)	
At least one previous prostate biopsy	43 (17.1)	53 (21.5)	0.26
Abnormal Digital Rectal Exam	7 (2.8)	70 (28.5)	<0.001
Family history of prostate cancer	28 (11.2)	62 (25.2)	<0.001
Prostate-specific antigen (ng/mL)	1.0 (0.1–8.4)	3.4 (0.0–93.8)	<0.001
Sarcosine uM in 20 uL serum (all samples)	16.2 (6.4–53.6)	15.8 (6.2–42.5)	0.40
Sarcosine (only PSA 2–10 ng/mL)	16.3 (8.1–37.6)	15.8 (6.2–42.5)	0.34
Gleason grade			N/A
5		7 (2.8)	
6		158 (64.2)	
7		56 (22.8)	
8		12 (4.9)	
9		8 (3.3)	
Missing		5 (2.0)	

**Fig. 1:**
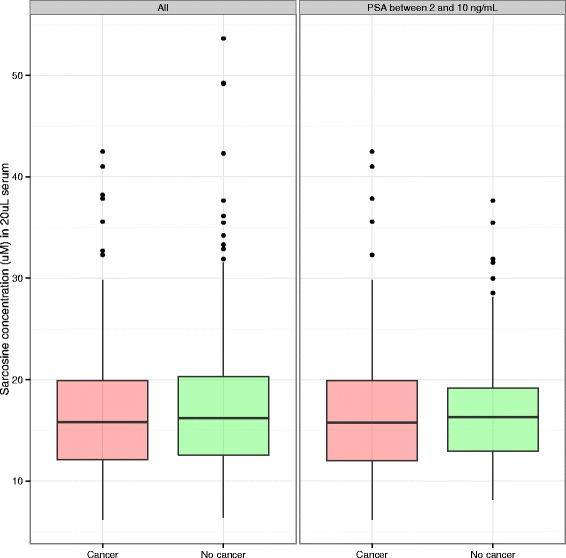
Boxplots of sarcosine measurements among cancer cases (*red*) and controls (*green*) in the SABOR study for all cases and controls (*left*, 246 cases, 251 controls) and those with PSA in the 2 to 10 ng/mL range (*right*, 188 cases, 53 controls)

Sarcosine levels were overlapping between the prostate cancer cases (median 15.8 uM, range 6.2 to 42.5 uM) and controls (median 16.2 uM, range 6.4 to 53.6 uM); see Fig. 1 and Table 1. The AUC of sarcosine was not statistically different from random chance either for participants with any PSA value (52.2 %) or those with PSA values in the range of 2 to 10 ng/mL (*n* = 53 controls, 188 cases, AUC 54.3 %). There was no independent predictive power of sarcosine for prediction of prostate cancer, controlling for PSA, DRE, and the other risk factors for prostate cancer (all *p* > 0.05, Table 2). Sarcosine similarly failed to be predictive of high- versus low-grade cancer (all *p* > 0.05).

**Table 2:** Multivariable logistic regression model results for prediction of prostate cancer detection based on sarcosine and standard risk factors

	Odds ratio (95 % confidence interval)	*P*-value
Age	1.0 (0.9–1.1)	0.11
Race		
African American	1.3 (0.6–3.0)	0.47
Other	1.2 (0.7–2.3)	0.49
At least one previous prostate biopsy	0.7 (0.3–1.3)	0.26
Abnormal Digital Rectal Exam	30.5 (11.8–92.2)	<0.001
Family history of prostate cancer	3.5 (1.8–6.8)	<0.001
Prostate-specific antigen (ng/mL)^a^	4.4 (3.3–5.9)	<0.001
Sarcosine uM in 20uL serum^a^	1.1 (0.7–1.8)	0.64

^a^Variable was log_2_ transformed in the model

## Discussion

Several studies have postulated that sarcosine is causally linked with the development and progression of prostate cancer. A large Norwegian study (*n* = 6000) concluded that sarcosine should be used as an early detection serum marker, but in contrast to other studies it found a decreased association with risk of prostate cancer rather than an increased one [3]. The reverse association could be due to the fact that baseline sarcosine measurements were used rather than those closer to the time of the biopsy as used in this study (92.7 % within 1 year prior to the biopsy, all the rest within 2.5 years prior). Two other studies, one from the US (*n* = 2234) and one from Italy (*n* = 602), found statistically significant increased associations between sarcosine and prostate cancer risk [4, 6]. The US study did not report an AUC for sarcosine, but rather reported its odds ratio with the outcome of prostate cancer to be 1.30 (95 % confidence interval 1.02 to 1.65). It noted that its inclusion in a clinical model did not improve the AUC. The Italian study investigated the AUC of sarcosine for all possible subsets of PSA, finding its max value of 64 % for use in men with PSA < 4 ng/mL, and its worst performance, which was no different from random chance, for men either with PSA > 10 ng/mL or between 4 and 10 ng/mL. The AUC remained marginally but statistically significantly better than chance for use among men with any value of PSA (AUC 57 %, 95 % CI 53 % to 61 %) as well as among men with PSA in the 2 to 10 ng/mL range (AUC 57 %, 95 % CI 52 % to 62 %).

The discordant findings of the SABOR prospectively-collected cohort with findings of some previous studies may have a number of explanations. First and foremost, previous studies noting an association between serum sarcosine and prostate cancer risk may be simply due to chance. The tendency to false positives is increased because of the multiple comparisons typically evaluated for sarcosine across different ranges of PSA; for example the Italian study reported 5 AUCs [6]. The previous US study did not report an AUC for sarcosine alone, but its inability to budge the AUC based on clinical risk factors in the study indicates the AUC may not have been statistically significantly different from 50 % in accordance with this study [4]. With the initial flurry of interest in this potential biomarker, publication bias may have led to submission and acceptance of only those studies that thereafter *confirmed* this association, meaning that other negative studies were not published. Other biases may also be operational, including the fact that few previous evaluations were conducted on prospectively-assembled cohorts of patients at risk of prostate cancer, or that the sarcosine measurements were taken too far in advance of the biopsy, as was the case for the large Norwegian study [3]. Unfortunately biomarker studies typically fail to report the time interval between biomarker measurement and the biopsy, which is particularly relevant for the prostate cancer cases. SABOR followed a rigorous protocol in which only cases were included where the biomarker was measured within a window of 2.5 years prior to the biopsy.

With its rigorous design, we believe this study eliminates serum sarcosine for further investigation as an early detection marker for prostate cancer. However, sarcosine may have a role in urine. Four studies investigating urine sarcosine for early detection all reported AUCs in the upper 60s and lower 70s, all statistically significantly better than chance in prediction [1, 7–9]. Urinary sarcosine seems to be more predictive of prostate cancer and may be utilized along with PSA and DRE in screening protocols.

## Conclusions

Serum sarcosine, while exhibiting promising results for the early detection of prostate cancer in in some prior studies, had no value in this independent analysis of men undergoing screening in San Antonio, Texas. Serum sarcosine should not be pursued further as a marker for the early detection of prostate cancer.

## Abbreviations

AUC: Area underneath the receiver-operating characteristic curve
DRE: Digital rectal examination
EDRN: Early Detection Research Network
GNMT: Glycine-N-methyltransferase
HR: Hazard ratio
OR: Odds ratio
PSA: Prostate-specific antigen
SABOR: San Antonio Biomarkers of Risk
US: United States
UTHSCSA: University of Texas Health Science Center at San Antonio
